# Metabolic and Nutritional Responses of Contrasting Aluminium-Tolerant Banana Genotypes Under Al Stress

**DOI:** 10.3390/plants14030385

**Published:** 2025-01-27

**Authors:** Xinran Wu, Shahbaz Khan, Yucheng Qi, Chuanling Zhang, Sumera Anwar, Liyan Yin, Jiaquan Huang

**Affiliations:** 1 School of Breeding and Multiplication, Sanya Institute of Breeding and Multiplication, College of Tropical Agriculture and Forestry, Hainan University, Sanya 572025, China; 22220951310015@hainanu.edu.cn (X.W.); shahbazbaloch@webmail.hzau.edu.cn (S.K.); 22220951310084@hainanu.edu.cn (Y.Q.); 2 Directorate of Agriculture Research (DAR) Uthal, Labella 69090, Balochistan, Pakistan; 3 School of Life Sciences, Hainan University, Haikou 570228, China; zcl@hainanu.edu.cn; 4 One Health Institute, Hainan University, Haikou 570228, China; 5 Department of Botany, Government College Women University Faisalabad, Faisalabad 38000, Pakistan; anwer_sumera@yahoo.com

**Keywords:** *Musa acuminata* L., aluminum tolerance, genotype-specific responses, root elongation, enzyme activity, nutrient retention

## Abstract

Aluminum (Al) toxicity is a major constraint to crop productivity in acidic soils, frequently encountered in banana-growing regions. This study investigates physiological and biochemical responses to Al stress in two Cavendish banana genotypes, Baodao and Baxi (*Musa acuminata* L.), which exhibit contrasting levels of Al tolerance. Banana plantlets were grown hydroponically under three AlCl_3_ concentrations (0, 100, and 500 μM) for 24, 48, and 72 h. Root elongation was progressively inhibited with increasing Al concentrations, with Baodao showing greater inhibition than Baxi. Al primarily accumulated in roots and displayed genotype-specific distribution patterns: Baodao concentrated more Al in root tips, suggesting lower exclusion efficiency. In contrast, Baxi, the Al-tolerant genotype, translocated Al from roots to shoots more effectively, indicating potential sequestration mechanisms in less sensitive tissues. Al stress influenced enzyme activities, with Baxi exhibiting higher phosphoenolpyruvate carboxylase and citrate synthase activities at 100 µM Al, while both genotypes showed similar reductions at 500 µM. Baodao experienced more pronounced reductions in H^+^-ATPase activity. At 100 µM Al, Baxi retained higher levels of key nutrients (P, Zn, Mg, Mn, Fe, K, and B) in essential tissues than Baodao. However, nutrient levels were reduced in both genotypes at 500 µM Al. These findings highlight Baxi’s superior resilience under Al stress, making it a suitable genotype for cultivation and breeding in acidic soils.

## 1. Introduction

Aluminum (Al) is the most abundant metal element in the Earth’s crust. When released in acidic soils, it converts into soluble Al^3+^ ions, the form most toxic to plants [1]. These ions bind with phosphates, forming insoluble compounds and reducing phosphorus (P) availability to plants [2]. In tropical regions, where soils are highly weathered, Al ions are particularly prevalent due to sustained high temperatures and frequent rainfall, which promote Al ion dissolution and soil acidity [3].

Banana (*Musa* spp.) cultivation plays a critical role in the economies of tropical and subtropical regions due to its importance in agriculture and trade. In addition to being a major source of carbohydrates, bananas are rich in potassium, phosphorus, and vitamins B and C, making them essential crops. China, the world’s second-largest banana producer, faces significant challenges from acidic soils in major banana-growing areas, including Hainan, Guangxi, Yunnan’s Hekou region, and Fujian’s Zhangzhou area [4,5]. In these areas, soil pH frequently falls below 5.5, which, combined with high nitrogen fertilization and subtropical climate, increases the risks of Al toxicity [6].

Al toxicity severely restricts banana cultivation by disrupting root function, nutrient uptake, and overall plant growth [7]. In acidic soils, Al displaces essential nutrients, such as potassium, calcium, and magnesium, reducing their bioavailability [8,9]. This nutrient displacement leads to imbalances that affect metabolic processes, further impeding plant development [10]. Such effects are exacerbated by tropical climates, which promote nutrient leaching and limit plant access to subsoil resources [10,11].

Upon exposure to Al, sensitive plant species show rapid physiological and biochemical responses. A common early symptom of Al toxicity is root growth inhibition, as Al binds to cell wall components and alters root structural integrity [10]. This response varies by genotype: in Al-sensitive plants, root elongation and cell wall composition are more adversely affected, with increased pectin and hemicellulose levels observed in the root elongation zone [12,13].

Plants that tolerate Al stress employ specific strategies to mitigate its toxic effects, such as limiting Al absorption or compartmentalizing it in less sensitive tissues [14,15]. For example, Al-tolerant plants can sequester Al in vacuoles or utilize organic acids to chelate Al ions, preventing them from damaging sensitive cellular sites [1,16]. Our previous analysis found that Al-tolerant banana genotypes can transport Al away from sensitive root tips, reducing damage and supporting overall growth [10].

While the effects of Al toxicity have been studied in various crop species, there is limited research on the specific physiological and metabolic responses of bananas, especially across genotypes with differing Al tolerance [7,17]. This study aims to address this gap by examining Al-tolerant and Al-sensitive banana genotypes over short-term exposure periods (24, 48, and 72 h) and assessing root elongation, nutrient redistribution, enzyme activity, and Al accumulation in different tissues. By detailing tissue-specific responses, particularly in root and shoot tissues, this research aims to provide insights into the mechanisms underlying Al tolerance in bananas, ultimately guiding breeding strategies for improved resilience in acidic soils.

The main hypothesis of this study is that Al-tolerant and Al-sensitive banana genotypes exhibit distinct patterns of Al uptake, nutrient balance, and metabolic adjustments under Al stress. We hypothesize that Al-tolerant genotypes will limit Al accumulation in sensitive tissues and maintain nutrient homeostasis more effectively than sensitive genotypes, thus sustaining better growth and metabolic function under Al exposure.

## 2. Results

### 2.1. Plant Growth After Al Treatment

The root growth of two banana genotypes, Baodao and Baxi, was assessed after 24, 48, and 72 h of exposure to various aluminum (Al) concentrations. Al treatment significantly impacted root elongation and relative elongation, revealing that increasing Al concentration suppressed root growth in both genotypes (Table 1). The duration of Al exposure significantly affected root elongation (*p* < 0.001) but was less impactful on relative elongation (*p* < 0.05). Genotypic differences were observed: Baodao showed greater sensitivity, with root elongation decreasing by 58% at 100 µM Al and 82% at 500 µM Al. Baxi exhibited a slight increase in root elongation at 100 µM Al after 24 h but decreased by 76% at 500 µM Al, mirroring Baodao’s response. This indicates genotype-specific responses to Al stress, particularly over time. Leaves showed no morphological changes after 72 h across all Al concentrations.

**Figure 1:**
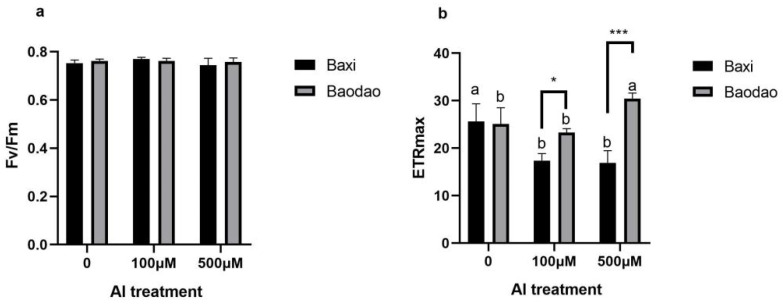
Chlorophyll fluorescence parameters of two banana genotypes after 24 h of exposure to 0, 100, and 500 µM Al in hydroponic culture: (**a**) maximum photosystem II efficiency (Fv/Fm) and (**b**) maximum electron transport rate (ETRmax). Different letters indicate significant differences between Al treatments within the same genotype; * and *** denote significant differences between genotypes at *p*-values of 0.05 and 0.001, respectively.

Maximum photosystem II efficiency (Fv/Fm), indicative of maximum photosynthetic efficiency, remained stable between genotypes (Figure 1). ETRmax varied significantly among genotypes and Al concentrations, reflecting differential photosynthetic performance under stress.

### 2.2. Al Uptake and Distribution in Banana Tissues

**Figure 2:**
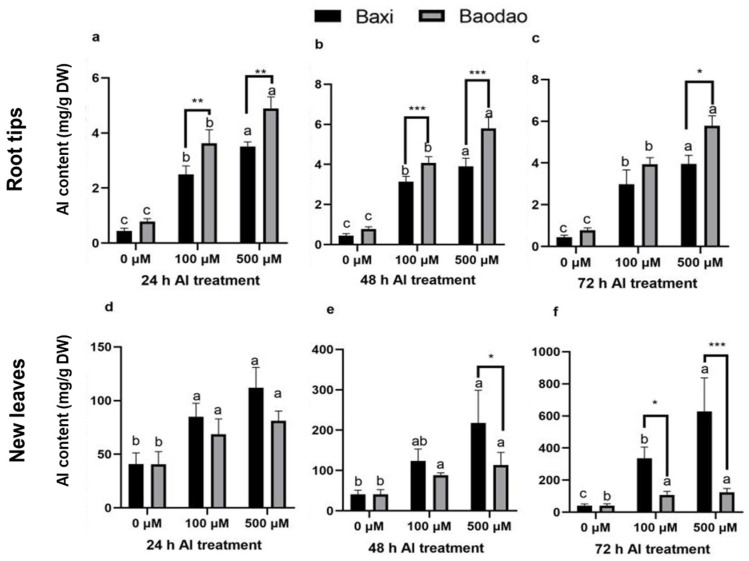

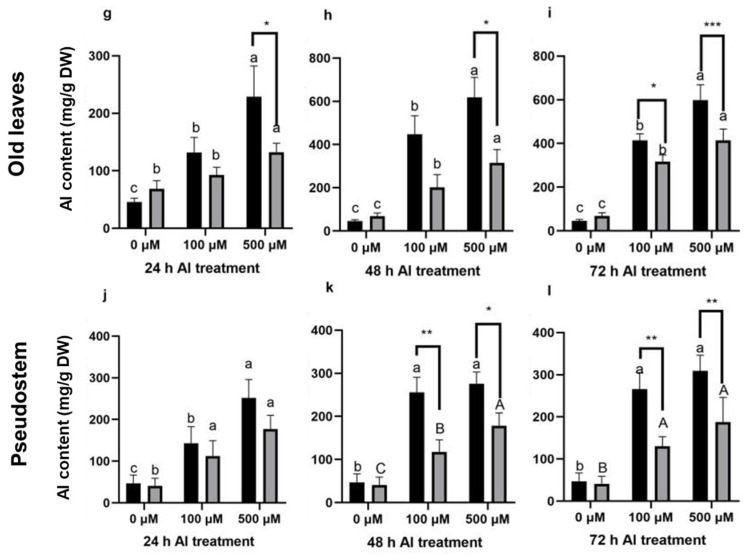
Al content in tissues of two banana genotypes after exposure to Al in hydroponic culture. Al content in root tips after 24 h (**a**), 48 h (**b**), and 72 h (**c**); Al content in new leaves after 24 h (**d**), 48 h (**e**), and 72 h (**f**); Al content in old leaves after 24 h (**g**), 48 h (**h**), and 72 h (**i**); and Al content in pseudostems after 24 h (**j**), 48 h (**k**), and 72 h (**l**). Different letters indicate significant differences between Al treatments within the same genotype, and *, **, and *** denote significant differences between genotypes at *p*-values of 0.05, 0.01, and 0.001, respectively.

Al content was measured in the root tips, new leaves, old leaves, and pseudostems of the two genotypes (Figure 2). Root tips consistently showed the highest Al content across all time points compared to other tissues. Prolonged Al exposure slightly increased Al levels in root tips but led to a more substantial accumulation in the leaves and pseudostems. Baodao accumulated significantly higher Al levels in root tips than Baxi, particularly at 24 and 48 h under 100 and 500 µM Al and at 72 h under 500 µM Al. In contrast, Baxi accumulated higher Al in the leaves and pseudostems than Baodao, indicating greater Al translocation capacity. This suggests that root tips are the primary site of Al toxicity, while Baxi’s tolerance may be linked to its ability to redistribute Al from roots to aerial parts.

**Figure 3:**
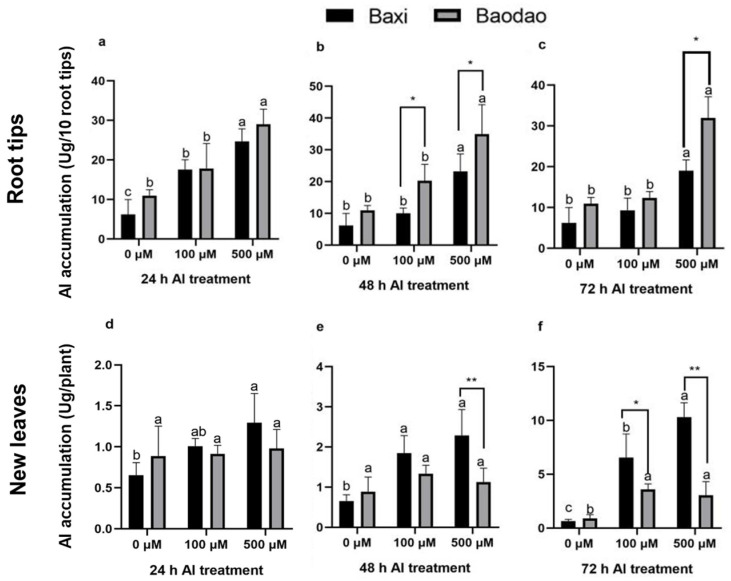

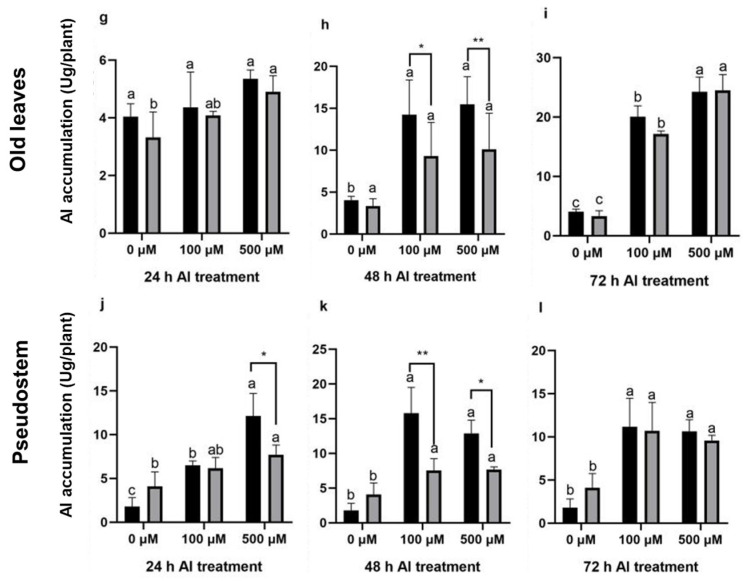
Al accumulation in tissues of two banana genotypes after exposure to Al in hydroponic culture. Al accumulation in root tips after 24 h (**a**), 48 h (**b**), and 72 h (**c**); Al accumulation in new leaves after 24 h (**d**), 48 h (**e**), and 72 h (**f**); Al accumulation in old leaves after 24 h (**g**), 48 h (**h**), and 72 h (**i**); and Al accumulation in pseudostems after 24 h (**j**), 48 h (**k**), and 72 h (**l**). Different letters indicate significant differences between Al treatments within the same genotype, and * and ** denote significant differences between genotypes at *p*-values of 0.05 and 0.01, respectively.

Over time, Al accumulation patterns diverged between the genotypes (Figure 3). Baodao retained more Al in root tips, whereas Baxi showed pronounced Al increases in pseudostems and leaves, particularly under prolonged exposure (48–72 h). For example, Baxi accumulated more Al in pseudostems after 48 h and in old leaves after 24 and 48 h under 500 µM Al. By 72 h, Al levels in old leaves of both genotypes significantly increased, with Baxi consistently surpassing Baodao. These findings highlight Baxi’s enhanced ability to translocate and sequester Al in aerial tissues as a potential mechanism of Al tolerance.

### 2.3. Enzyme Activities Related to Carbon Metabolism in Root Tips

Aluminum (Al) treatment significantly influenced the activities of key enzymes involved in organic acid formation and carbon metabolism in banana root tips, including phosphoenolpyruvate carboxylase (PEPC), citrate synthase (CS), malate dehydrogenase (MDH), plasma membrane H+-ATPase, and glycolate oxidase (GO).

**Figure 4:**
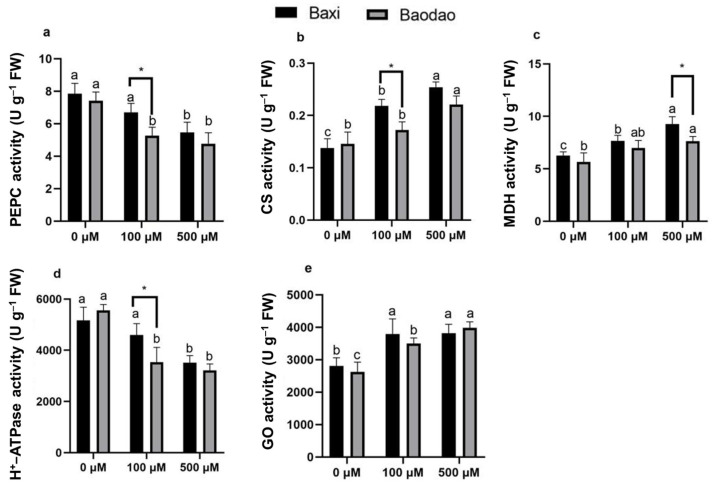
Enzyme activity of carbon metabolism in root tips of two banana genotypes after exposure to Al in hydroponic culture. (**a**) Phosphoenolpyruvate carboxylase (PEPC), (**b**) citrate synthase (CS), (**c**) malate dehydrogenase (MDH), (**d**) plasma membrane H^+^-ATPase, and (**e**) glycolate oxidase (GO). Different letters indicate significant differences between Al treatments within the same genotype, and * denotes significance at a *p*-value of 0.05 between the two genotypes.

Al exposure reduced PEPC activity in both genotypes, with Baxi maintaining higher activity than Baodao at 100 µM Al, indicating greater tolerance to moderate Al stress (Figure 4a). However, at 500 µM Al, both genotypes experienced similar reductions, suggesting that high Al levels overwhelm their defense mechanisms.

Al increased CS activity in both genotypes, with greater enhancements in Baxi compared to Baodao. At 100 µM Al, Baxi exhibited a 58% increase versus Baodao’s 18% increase. At 500 µM Al, increases were 84% for Baxi and 51% for Baodao (Figure 4b). The differences between genotypes were more pronounced at lower Al concentrations.

MDH activity, initially low under control conditions, increased significantly with Al exposure. At 100 µM Al, Baxi and Baodao showed rises of 23.8% and 22.4%, respectively, compared to the control. At 500 µM Al, the increases were 35.4% for Baxi and 47.9% for Baodao (Figure 4c).

Al significantly reduced H^+^-ATPase activity, with Baodao experiencing sharper declines (36% and 42% at 100 and 500 µM Al, respectively) compared to Baxi (11% and 32%) (Figure 4d). This indicates a better capacity in Baxi to maintain cellular ion balance under Al stress.

GO activity increased in response to Al in both genotypes, with Baodao showing higher increases at 500 µM Al (52%) compared to Baxi (35.9%) (Figure 4e). At 100 µM Al, Baxi exhibited a slightly greater increase (35.1% vs. 33.5%), suggesting differential responses depending on Al concentration. These findings highlight genotype-specific enzyme activity changes, with Baxi demonstrating a more robust enzymatic response under moderate Al stress, which could potentially contribute to its enhanced Al tolerance.

### 2.4. Primary Metabolites Alteration

#### 2.4.1. Soluble Protein Content

**Figure 5:**
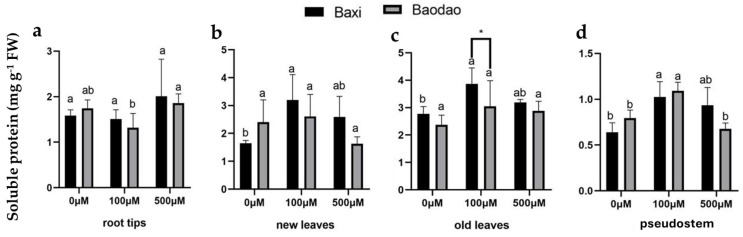
Soluble protein content in various tissues of two banana genotypes after 24 h of Al exposure in hydroponic culture: (**a**) root tips, (**b**) new leaves, (**c**) old leaves, and (**d**) pseudostems. Different letters on bars (*n* = 3 ± SE) indicate significant differences between Al treatments within the same genotype, and * indicates a significant difference between genotypes at a 0.05 probability level.

Soluble protein content exhibited variable patterns across organs and Al treatments. In root tips, it slightly increased after 500 µM Al exposure in both genotypes (Figure 5a). In Baxi, soluble protein levels in both new and old leaves were under 100 µM Al, whereas Baodao showed no significant changes (Figure 5b,c). In pseudostems, the soluble protein content increased at 100 µM Al but decreased at 500 µM Al in both genotypes (Figure 5d).

#### 2.4.2. Carbohydrates

**Figure 6:**
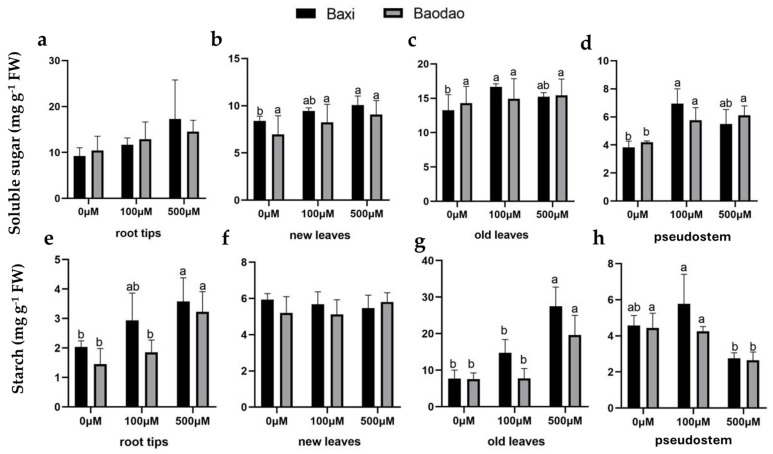

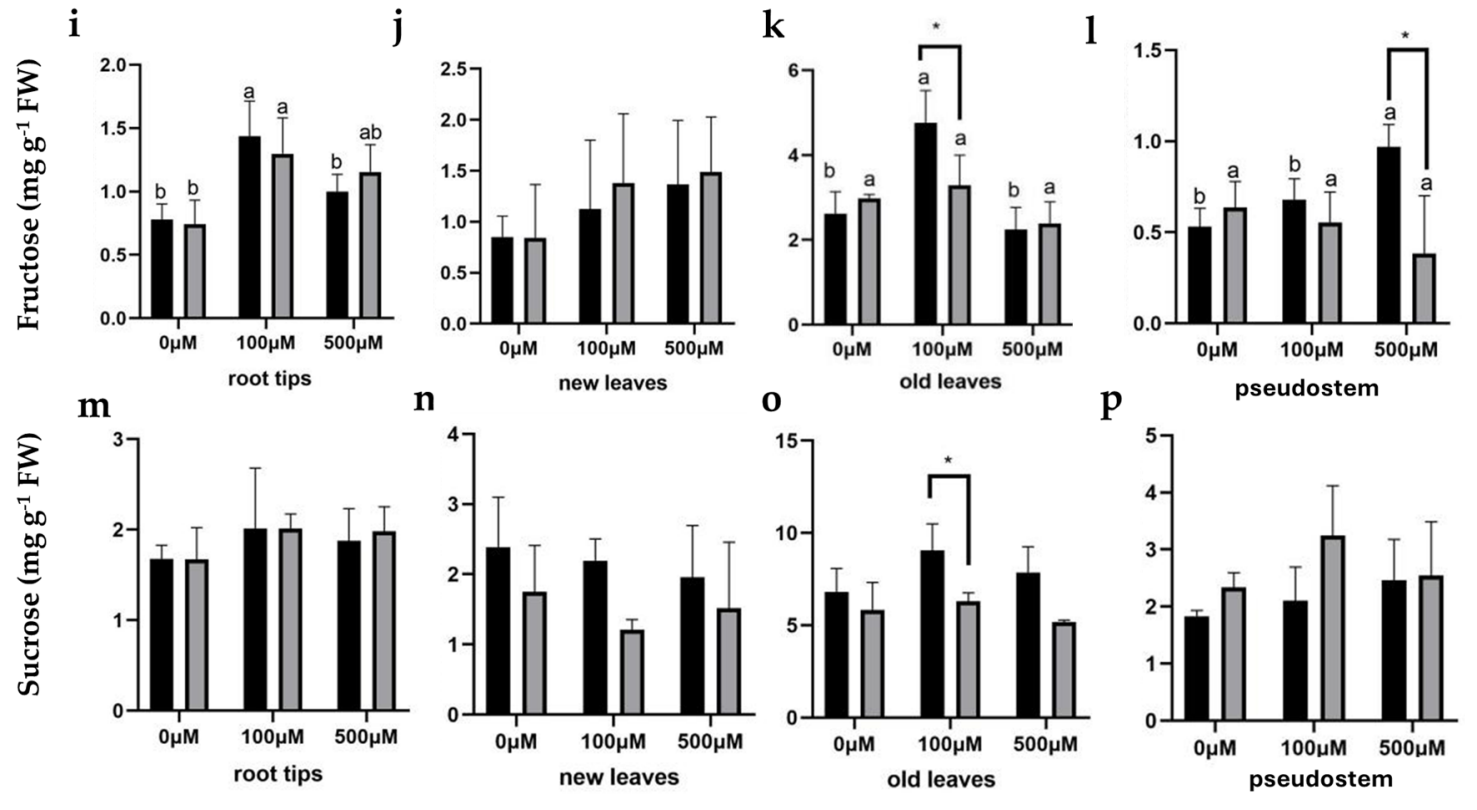
Carbohydrate content in different tissues of two banana genotypes after 24 h of Al exposure in hydroponic culture. Soluble sugar (**a**–**d**), starch (**e**–**h**), fructose (**i**–**l**), and sucrose (**m**–**p**) levels in root tips, new leaves, old leaves, and pseudostems following 0, 100, and 500 µM Al exposure. Different letters indicate significant differences between Al treatments within the same genotype, and * denotes significance between the genotypes at a *p*-value of 0.05. Asterisks indicate significant differences between genotypes.

Carbohydrate responses paralleled soluble protein content trends, with limited differences between genotypes. Soluble sugar content in root tips remained unchanged under Al stress (Figure 6a). In Baxi, new leaves showed increased soluble sugar levels at 500 µM Al, while Baodao was unaffected (Figure 6b,c). In pseudostems, soluble sugar content significantly increased at 100 µM Al in both genotypes (Figure 6d).

Starch content increased in root tips and old leaves under Al exposure for both genotypes, while new leaves were unaffected (Figure 6e–g). Starch levels in pseudostems decreased only under 500 µM Al (Figure 6h).

Fructose content in root tips significantly increased at 100 µM Al in both genotypes but decreased at 500 µM Al compared to 100 µM (Figure 6i). In new leaves, fructose levels were unaffected (Figure 6j). In old leaves, fructose increased significantly at 100 µM Al in Baxi but not in Baodao, with levels returning to control values at 500 µM Al (Figure 6k). Pseudostems of Baxi showed increased fructose at 500 µM Al, while Baodao remained unchanged (Figure 6l).

Sucrose content was unaffected by Al exposure in all tissues of both genotypes (Figure 6m–p). These findings highlight tissue- and genotype-specific variations in primary metabolite responses to Al stress, with Baxi showing greater metabolite adjustments under moderate Al levels, potentially contributing to its higher Al tolerance.

### 2.5. Mineral Nutrient Content Changes in the Tissues of Banana Genotypes with Al Treatment

Al exposure significantly influenced the mineral nutrient content in different tissues of Baodao and Baxi. Nutrient content at 0 h was consistent across tissues and was used as a control. Al significantly reduced P content in all tissues, with higher reductions at 500 µM Al, especially in Baodao. Baxi maintained higher P levels than Baodao in roots, old leaves, and pseudostems, particularly under 100 µM Al (Table S1). Al exposure time further influenced P levels, notably in new and old leaves.

Zn content was significantly affected in roots, old leaves, and pseudostems. Baxi exhibited higher Zn levels than Baodao in roots and old leaves, especially at 100 µM Al (Table S1). Both Al and genotype significantly impacted the Mg content in roots and old leaves, with Baxi maintaining higher levels, particularly under 100 µM Al (Table S2). Higher Al concentrations (500 µM) caused significant Mg reductions, especially in roots. Exposure time significantly influenced Mg levels in new leaves.

Al affected Mn content in all tissues. Baxi showed higher Mn levels, particularly in old leaves and pseudostems, under both 100 µM and 500 µM Al (Table S2). Al exposure significantly impacted Fe content in all tissues. Baxi exhibited higher Fe levels than Baodao in roots, particularly under Al stress. Time of Al exposure notably affected Fe levels in new leaves and pseudostems (Table S3).

Al significantly reduced K content across all tissues, especially in old leaves. Baxi had consistently higher K levels than Baodao, particularly in roots and old leaves (Table S3). B content was significantly higher in Baxi’s new and old leaves compared to Baodao, particularly under 100 µM Al. Baxi showed sharp B increases in old leaves at 48 h with 100 µM Al, while Baodao remained stable (Table S4). Al exposure significantly reduced Ca levels in roots and leaves, with reductions increasing with higher Al concentrations and longer exposure times. Baxi generally retained higher Ca levels in roots and leaves than Baodao (Table S4). These results highlight genotype-specific differences in mineral nutrient responses to Al stress, with Baxi demonstrating greater resilience and nutrient retention, particularly under moderate Al concentrations.

### 2.6. Heat Map of the Carbohydrate Metabolism Enzymes, Metabolites, and Root Elongation

**Figure 7:**
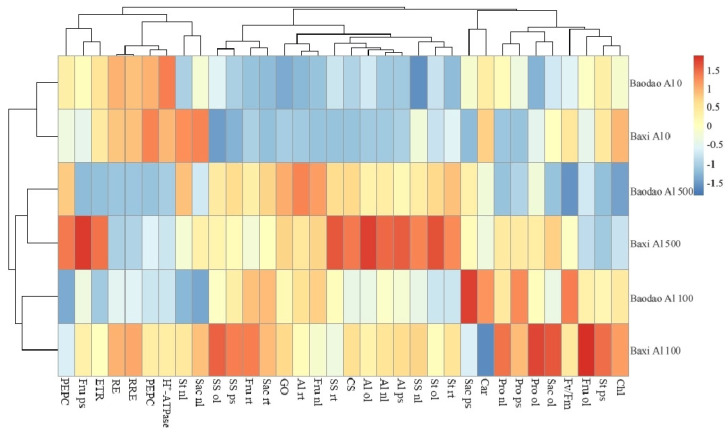
Cluster heatmap of root elongation, Al accumulation, carbon metabolism enzymes, chlorophyll fluorescence, and metabolite levels. Abbreviations: Chl—chlorophyll content, St—starch, Sac—sucrose, Pro—total soluble protein, Car—carotenoids, SS—soluble sugar, Al—aluminum, CS—citrate synthase, Fru—fructose content, GO—glycolate oxidase, PEPC—phosphoenolpyruvate carboxylase, MDH—malate dehydrogenase, ps—pseudostem, ol—old leaf, nl—new leaf, rt—root tip, and ETR—electron transport rate.

Baxi and Baodao at 100 µM Al clustered together, followed by a clustering of all Al treatments. This pattern showed that both genotypes responded similarly to Al exposure, and Al treatment at both 100 and 500 µM greatly changed the physiological profile in bananas (Figure 7). Without Al, the Al content, root elongation rate, and carbohydrate metabolism enzymes were similar in both genotypes. At 100 µM Al, Baxi showed higher root elongation, relative root elongation, starch content in the roots and pseudostems, fructose in roots and old leaves, sucrose content in old and new leaves, protein content in new and old leaves, and soluble sugar in the pseudostems, old leaves, and new leaves. In contrast, Baodao had higher carotenoids in mature leaves and sucrose in the pseudostems. At 500 µM Al, Baxi showed higher Al content in pseudostems, new leaves, and old leaves, starch in roots, old leaves, and new leaves, soluble sugar in new leaves and roots, protein in roots, pseudostems, and new leaves, fructose in pseudostems, CS activity, and ETR. In contrast, Baodao had higher Al in roots, starch in roots, and fructose in new leaves.

### 2.7. Correlation of Mineral Nutrient Content in Tissues

**Figure 8:**
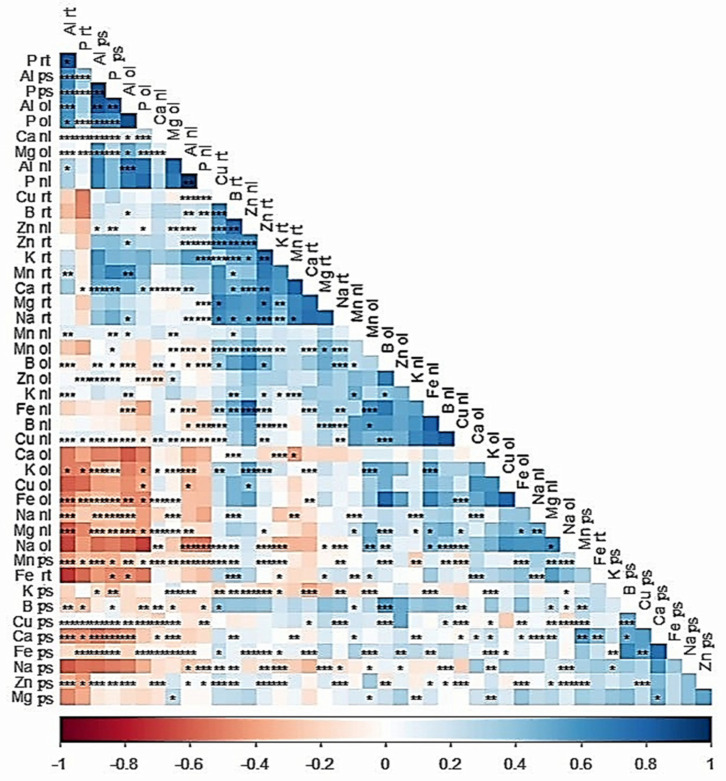
Correlation among mineral nutrient contents (Al, P, Ca, K, Zn, Fe, Cu, Mn, B, and Na) in pseudostems (ps), root tips (rt), new leaves (nl), and old leaves (ol) of banana plantlets. *, **, and *** denote significance at *p*-values of 0.05, 0.01, and 0.001, respectively.

The Al content in the root tips, old leaves, new leaves, and pseudostems was negatively related to most of the mineral nutrients (Figure 8). For example, the Al content in the root tips and pseudostems was negatively associated with the K in the old leaves, Fe in old leaves, Na in new leaves, Mg in new leaves, and Mn, B, Cu, Ca, and Zn in the pseudostems. At the same time, Al in roots and pseudostems was positively correlated with the Al in the old leaves and P in roots, pseudostems, and old leaves. Similarly, Al content in new leaves was positively correlated to the P in new leaves. In contrast, Cu in the roots, B in the roots, Zn in new leaves and roots, and K in the roots showed positive relationships with Al content.

## 3. Discussion

The prevalence of Al in China’s acidic soils, particularly in banana orchards, poses a significant challenge to sustainable production. Understanding how different banana genotypes respond to Al exposure at metabolic and nutrient levels is essential for mitigating its adverse effects and enhancing crop resilience.

### 3.1. Differential Responses of Badao and Baxi Genotypes to Al Stress

Relative root elongation is a well-established indicator for assessing Al tolerance in plants. Observing root elongation under Al exposure offers insights into the resilience of different genotypes [10]. Our data reveal that both Al concentration and exposure duration markedly affect root elongation and relative elongation in both genotypes, underscoring the detrimental influence of Al on root growth. Specifically, prolonged Al exposure exacerbates root growth inhibition, particularly at higher concentrations, suggesting a cumulative impact of Al on root cells over time. Mechanistically, approximately 90% of Al^3+^ ions bind to the cell wall, particularly to the pectin component, where negatively charged carboxylic groups attract Al ions. This Al binding decreases the cell wall’s flexibility and enzymatic function, restricting root elongation. Previous research has linked Al-induced root growth inhibition to disruptions in cell cycle progression [18], reduced mitotic and meiotic activity [19], and impaired microtubule formation [20].

In this study, Baodao and Baxi displayed contrasting responses to Al, suggesting genotype-dependent Al tolerance mechanisms. Baodao’s root elongation was notably suppressed under both 100 µM and 500 µM Al, indicating higher sensitivity to Al stress. In contrast, Baxi exhibited initial root elongation stimulation at 100 µM Al, followed by stabilization, suggesting an adaptive response to moderate Al levels. This difference in root elongation responses highlights genetic variation in Al tolerance, underscoring the potential for breeding bananas with enhanced resilience to acidic soils.

### 3.2. The Al-Sensitive Genotype Accumulates More Al in Root Tips with Limited Translocation

Al accumulation dynamics in Baodao (Al-sensitive) and Baxi (Al-tolerant) were examined across varied concentrations and durations of Al exposure. Th results showed a hierarchical Al distribution: root tips displayed the highest Al levels, followed by pseudostems, while new and old leaves had a relatively lower Al content. Interestingly, while old leaves accumulated Al over 24 and 48 h, new leaves eventually matched or exceeded this accumulation by 72 h, suggesting progressive translocation of Al from roots to shoots. Across tissues and time points, Al content generally increased with concentration, especially from 24 to 48 h in root tips, before plateauing at 72 h. This pattern underscores the role of root tips as primary sites for Al accumulation and transport initiation, with subsequent movement toward aerial parts [21,22]. Al’s detoxification and storage in leaves are likely mediated by vacuolar sequestration or organic acid binding [15,23].

Baodao consistently accumulated higher Al levels in roots, especially under elevated Al conditions, highlighting its sensitivity at the root tips, where Al binding is most pronounced [24]. In contrast, Baxi demonstrated efficient Al translocation to pseudostems, new leaves, and old leaves, suggesting that it manages Al stress more effectively through redistribution and sequestration mechanisms [1]. This variation suggests that Baodao may retain more Al in cell walls, whereas Baxi’s Al translocation indicates potential detoxification adaptations.

Our findings indicate that the Al-tolerant genotype, Baxi, employs distinct transporter mechanisms to translocate and sequester Al. Genotypic differences exist in the effective Al management through root-to-shoot translocation and sequestration transporters that were likely upregulated in Baxi. Studies have shown the involvement of Al transporter proteins, such as Nrat1 and AtNIP1;2, in Al uptake, translocation, and vacuolar storage, potentially decreasing toxicity [25,26]. In contrast, mutants deficient in these transporters exhibit reduced translocation and elevated Al retention in root cell walls, which increases their sensitivity to Al toxicity. Additionally, vacuolar and plasma membrane transporters, such as HmVALT1 and HmPALT1, are associated with vacuolar Al sequestration, crucial for Al tolerance [27].

In both genotypes, Al accumulated steadily in root tips over time, but Baodao generally retained higher Al levels in roots, indicating limited Al exclusion capacity [10,28]. In pseudostems, both genotypes showed increased Al accumulation with time, although Baxi had a pronounced increase after 48 h at elevated Al levels, suggesting a more active long-term response. Baodao’s minimal Al accumulation in old leaves at 24 and 48 h contrasts with Baxi’s robust increase under higher Al exposure, suggesting differential detoxification or adaptive mechanisms [1]. By 72 h, both genotypes showed increased Al in pseudostems, yet Baxi’s more rapid translocation emphasizes its ability to manage Al in aerial tissues, reducing root toxicity.

### 3.3. Tolerant Genotype Showed More Efficient and Regulated Metabolic Adjustment

Phosphoenolpyruvate carboxylase (PEPC) is key in C4 plants for primary atmospheric CO_2_ fixation and organic acid biosynthesis, converting phosphoenolpyruvate (PEP) into oxaloacetate through irreversible carboxylation. In C3 species, such as banana plants, PEPC is used differently and assists in replenishing tricarboxylic acid (TCA) cycle intermediates and pH regulation by generating organic acids, such as malate, particularly under stress conditions [29,30]. Similarly, in non-photosynthetic tissues, like root tips, PEPC ensures adequate ATP production in mitochondria to support root growth and cellular activity [31]. In our study, PEPC activity in the root tips of banana genotypes declined in both genotypes as Al concentration increased. However, at moderate Al levels (100 µM), the Al-tolerant genotype Baxi maintained higher PEPC activity than Baodao, suggesting more effective carbon fixation and a greater resilience to Al toxicity [32,33]. At higher Al levels (500 µM), both genotypes exhibited similar reductions in PEPC activity, likely due to cellular damage, such as membrane and chloroplast disruptions, or shifts in metabolic focus under severe stress [20]. These findings imply that while Baxi can maintain higher PEPC activity under moderate stress, this advantage diminishes with increasing Al toxicity, likely due to energy constraints under extreme conditions. Previous studies in soybean and common beans indicate a correlation between higher PEPC expression and Al tolerance in resistant genotypes [34,35].

Malate dehydrogenase (MDH) and citrate synthase (CS), enzymes central to the TCA cycle, play essential roles in Al tolerance. CS initiates the TCA cycle, and MDH catalyzes the conversion of malate to oxaloacetate while contributing to cellular redox balance through the malate–aspartate shuttle [36]. Under Al stress, both genotypes showed an increase in MDH activity, with a more substantial rise in Baxi, particularly at 500 µM Al. This upregulation in MDH may help synthesize malate to counter Al toxicity and oxidative stress [37]. While Baodao’s marked increase at high Al concentrations may reflect a stress response, Baxi’s controlled response suggests efficient metabolic regulation, aligning with its higher tolerance to Al. At moderate Al levels, Baxi also exhibited a more substantial increase in CS activity compared to Baodao, enhancing its Al detoxification through citrate binding [38]. At higher Al levels, both genotypes showed similar CS activity, indicating a saturation point in metabolic adjustments. Baxi’s enhanced CS response at moderate Al levels likely contributes to its superior Al tolerance compared to Baodao, which displayed a weaker metabolic response under similar conditions.

Organic acids, such as malate, citrate, succinate, and oxalate, play vital roles in detoxifying Al by forming non-toxic organic acid–Al^3+^ complexes, primarily in the rhizosphere, preventing Al uptake [1]. Within plant cells, Al detoxification occurs via internal malate and citrate, which sequester Al in vacuoles [38]. MDH activity supports this process, enhancing cellular energy production, redox balance, rhizosphere pH regulation, and overall tolerance to Al stress [35]. Studies have shown that increased MDH and CS expression can elevate malate and citrate production, bolstering Al resistance [39,40]. Even with reduced PEPC activity under Al stress, heightened MDH and CS activities ensure adequate organic acid production, prioritizing pathways that detoxify Al [41].

H^+^-ATPase activity is also essential for maintaining proton gradients and cellular pH under stress, as well as supporting Al-induced citrate efflux for detoxification [42]. Al exposure significantly reduced H^+^-ATPase activity in both genotypes, with a more substantial reduction in Baodao, indicating its higher sensitivity to Al toxicity. Baxi’s relatively stable H^+^-ATPase activity at 100 µM Al suggests better pH regulation and ion transport under moderate stress [43], contributing to its superior Al tolerance.

Glycolate oxidase (GO), localized in the peroxisome, plays a role in the photorespiratory pathway, converting glycolate to glyoxylate and producing H_2_O_2_ as a byproduct [44]. GO plays a role in oxalate accumulation in plants. Both genotypes showed increased GO activity under Al stress, likely reflecting an adaptive response to oxidative stress [45]. This increase may signal ROS-mediated defense activation. Baxi’s general resilience may involve more effective ROS management, whereas Baodao’s increased photorespiration could represent a compensatory response to greater oxidative damage.

The differential responses to Al stress across banana genotypes and tissues highlight distinct mechanisms governing carbohydrate stability and metabolism. Root tips demonstrated minimal changes in soluble proteins, soluble sugars, and sucrose under Al exposure, indicating potential reliance on exclusion mechanisms or sequestration strategies to mitigate toxicity. Furthermore, the sucrose content remained unaffected across all tissues. Sucrose is the primary form of carbohydrate transported in the phloem. Its stability under Al stress may indicate that sucrose transport and allocation mechanisms are preserved despite stress conditions, preventing significant depletion or accumulation in root tips [46].

In contrast, pseudostems and leaves exhibited significant metabolic shifts, particularly in the Al-tolerant genotype, Baxi. This suggests a tissue-specific regulation of carbohydrate metabolism, potentially involving both reduced carbohydrate utilization due to lower growth rates and changes in sugar transport dynamics via the phloem.

The increase in soluble sugar content observed in Baxi’s new leaves and pseudostems under moderate Al levels aligns with its enhanced adaptability [47]. These sugars may act as osmoprotectants or antioxidants, mitigating oxidative stress induced by Al exposure [48,49]. Moreover, starch accumulation in roots and old leaves under Al stress could indicate inhibited carbohydrate utilization, as reduced root elongation likely diminishes metabolic demand. Notably, the rise in fructose content in root tips at low Al concentrations may reflect enhanced invertase activity, converting sucrose into glucose and fructose to support energy demands or signaling pathways under stress [50].

The role of phloem transport in carbohydrate redistribution during Al stress warrants further consideration. Al exposure may alter the dynamics of carbohydrate loading and unloading in the phloem, leading to tissue-specific differences in sugar accumulation. In Baxi, increased sugar levels in leaves and pseudostems may result from more efficient carbohydrate translocation, facilitating their role in osmotic adjustment and stress mitigation [48,49,50,51]. In contrast, the relatively unchanged sucrose content across tissues suggests that sucrose transport mechanisms remain stable, possibly preserving energy reserves for critical metabolic functions.

The observed reduction in starch content in pseudostems at higher Al concentrations suggests a shift in carbohydrate allocation, potentially prioritizing soluble sugars for immediate stress responses over storage forms. This dynamic response underscores the complexity of metabolic adjustments in Al-tolerant genotypes, like Baxi, which exhibit greater flexibility in reallocating resources to maintain growth and mitigate stress effects [21,48,52].

In summary, the interplay between reduced carbohydrate utilization due to lower growth rates and altered phloem transport likely contributes to the observed tissue-specific carbohydrate profiles under Al stress. These findings underscore the importance of integrated metabolic and transport mechanisms in determining Al tolerance, particularly in genotypes, like Baxi, which demonstrate superior adaptive capacity through regulated carbohydrate metabolism and redistribution.

### 3.4. Tolerant Genotype Modulates ETRmax as a Protective Strategy Without Compromising PSII Efficiency

The Fv/Fm ratio, representing the maximum quantum efficiency of photosystem II (PSII), remained stable under Al exposure in both genotypes, indicating no significant impairment in PSII function across the tested Al concentrations (100 µM and 500 µM). This stability suggests that Al stress, within the exposure levels and duration used, did not directly impact the structural integrity of the PSII complex or disrupt its light-harvesting capacity. The absence of change in Fv/Fm implies that the oxidative and metabolic challenges induced by Al do not interfere directly with PSII’s energy capture and transfer efficiency [48,49]. In contrast, the maximum electron transport rate (ETRmax) exhibited differential responses between the two genotypes, highlighting distinct adaptation strategies under Al stress. The tolerant genotype, Baxi, showed a controlled reduction in ETRmax, which likely acts as a defensive adjustment to limit reactive oxygen species (ROS) accumulation and conserve energy resources in response to Al exposure. This strategic downregulation appears to aid Baxi in maintaining PSII efficiency without escalating ROS production, thus enhancing its overall stress tolerance. In contrast, the sensitive genotype, Baodao, displayed an increase in ETRmax at high Al concentrations, indicating a compensatory response. This increase, however, may not confer stress tolerance; rather, it suggests an overcompensatory attempt to counterbalance the adverse effects of Al, potentially exacerbating oxidative damage instead of effectively mitigating it [53,54]. These findings underline Baxi’s ability to modulate the photosynthetic electron transport rate in a controlled fashion under Al stress, avoiding detrimental impacts on PSII efficiency, while Baodao’s lack of regulation in ETRmax may contribute to its greater sensitivity to Al toxicity.

### 3.5. Prolonged Al Exposure and Increased Concentration Induce Greater Nutrient Imbalance in the Sensitive Banana Genotype

Al exposure significantly impacted nutrient accumulation in two banana genotypes, Baxi and Baodao, with distinct responses observed across various tissues. At a lower Al concentration (100 µM), both genotypes initially showed increased accumulation of P, Zn, K, and Mg, particularly in the pseudostem and leaves. This suggests that the 72 h exposure to 100 µM Al was insufficient to induce nutrient reduction. Previous studies have reported nutrient imbalances or even increases at low Al concentrations or during short-term exposure [55,56]. For example, Silva et al. [57] reported no reduction in root length with a reduction in Ca and Mg uptake at 100 µM, while Lidon et al. [58] observed increased uptake of K, Ca, Mg, and Fe at 0.33 mg/L Al, which then decreased at higher concentrations. However, when the Al concentration was increased to 500 µM or the exposure time was prolonged, a significant reduction in nutrient levels was observed across most tissues, showing a time- and concentration-dependent decrease in nutrients. This reduction is attributed to competition between Al and nutrients for root exchange sites, leading to reduced or imbalanced nutrient accumulation [11,18]. A comparison of various tissues and time points revealed that Baxi exhibited greater resilience and adaptive responses in nutrient content under Al stress compared to Baodao. Specifically, under 100 µM Al exposure, Baxi maintained higher levels of P, Zn, Mg, Mn, Fe, K, and B in critical tissues, such as roots, leaves, and pseudostems. However, both genotypes showed reductions in P, Mg, and K levels as Al concentrations increased to 500 µM, with Baodao experiencing more pronounced decreases.

The correlation matrix (Figure 8) further elucidates the relationship between Al content and mineral nutrients. Al content in root tips, leaves, and pseudostems showed a negative correlation with key nutrients, including K, Mg, Mn, B, Cu, Ca, and Zn. This indicates that Al exposure disrupts nutrient uptake and distribution. Interestingly, Al content in roots and pseudostems was positively correlated with P in roots, pseudostems, and leaves, as well as Al content in old leaves. These positive correlations suggest that P plays a role in Al stress adaptation, potentially aiding in Al detoxification or compartmentalization [59,60].

Zn and Mn content initially increased in both genotypes, particularly at 24 h of exposure, before declining over time. Fe content also notably increased with Al exposure, while B content in Baxi peaked under stress conditions. These findings are consistent with previous studies suggesting that Al-tolerant genotypes can maintain nutrient content under Al exposure [18,56,61,62]. The lower nutrient imbalance in tolerant genotypes is directly linked to the lower Al levels in their roots, achieved by excluding Al from roots or by transporting and sequestering Al in the other tissues [18,56].

The differential nutrient accumulation between Baxi and Baodao highlights Baxi’s more effective mechanisms for nutrient transport, sequestration, and homeostasis under Al stress. The ability of Baxi to maintain higher levels of P, Zn, and Mg, especially at 100 µM Al, suggests that it possesses more robust nutrient transporters and a greater capacity to detoxify and sequester Al [56]. This capability helps protect nutrient uptake and transport systems. In contrast, Baodao, which exhibited more significant nutrient reductions at higher Al concentrations, may lack the same efficiency in nutrient transport and Al sequestration. This deficiency likely leads to reduced nutrient availability over time. The roots and new leaves of Baodao were the most affected tissues, with substantial reductions in nutrient content, indicating disrupted nutrient uptake and translocation under Al stress [61,63]. The inability to regulate nutrient levels effectively in these critical tissues contributes to Baodao’s heightened sensitivity to Al toxicity. These findings underscore the importance of genotypic differences in Al tolerance mechanisms, particularly the role of nutrient transporters and sequestration pathways in alleviating the adverse effects of Al on nutrient homeostasis.

## 4. Materials and Methods

### 4.1. Plant Materials and Culture Conditions

Two banana genotypes, Baodao and Baxi, triploid (AAA) Cavendish cultivars of *Musa acuminata* L., were obtained from the Banana Rapid Propagation Base in Danzhou, Hainan, China. These genotypes were selected based on their differential tolerance to aluminum (Al), as observed in our prior study [10]. Healthy seedlings were selected, their roots were thoroughly cleaned, and they were subsequently transferred to a 1/5 Hoagland hydroponic nutrient solution in aerated plastic containers. The cultivation took place in a greenhouse at a constant temperature of 26 °C under a 16 h light/8 h dark photoperiod with an average humidity maintained at 75–80%. The nutrient solution was replaced every three days.

Following a three-week pre-culture period, seedlings exhibiting three leaflets and one new emerging leaf were transferred to a CaCl_2_ solution (pH 4.5, 9 plants per container) for 24 h to acclimate to acidic conditions. The seedlings were then exposed to solutions containing 0, 100, and 500 μM AlCl_3_ in addition to 0.5 mM CaCl_2_ and 1/5 modified Hoagland solution for 24, 48, and 72 h. Each treatment was replicated at least three times.

Samples for biochemical assays and Al content and Al accumulation were collected from the roots, new leaves, old leaves, and pseudostems. The pseudostem in a banana refers to a false stem structure formed by tightly packed and overlapping leaf sheaths.

### 4.2. Root Length and Elongation Rate Measurement

Root lengths were measured prior to and after 24, 48, and 72 h of Al exposure. Relative root elongation was calculated as a percentage of root growth under Al exposure compared to the control (no Al), based on the previously established protocol [10].

### 4.3. Carbon Metabolism Enzyme Activity Assay

Root tips from 18 banana seedlings were collected to assess the activities of key enzymes involved in carbon metabolism. Enzyme activities for glycolate oxidase (GO), citrate synthase (CS), phosphoenolpyruvate carboxylase (PEPC), hydrogen ATPase (H^+^-ATPase), and malate dehydrogenase (MDH) were quantified using the kits from Jiangsu Meimian Industrial Co., Ltd., Changzhou, China. Optical density (OD) values, indicative of absorbance at specified wavelengths, were measured and converted to enzyme activity according to the manufacturer’s instructions using a spectrophotometer (UV-1800, Shimadzu Corporation, Kyoto-Shi, Japan).

### 4.4. Soluble Protein Content Assay

The soluble protein in root tips, leaves, and pseudostems was determined using the Coomassie Brilliant Blue method, following Bradford’s protocol [64]. Approximately 0.1 g of plant tissue (leaves, stems, or root tips) was ground with 1.5 mL of 0.05 M phosphate buffer for protein extraction. After homogenization and centrifugation, the supernatant was collected, and the protein concentration was measured by reacting 20 μL of the sample with 200 μL of Coomassie Brilliant Blue. The absorbance was measured at 595 nm using a spectrophotometer (UV-1800, Shimadzu Corporation, Kyoto-Shi, Japan), and the protein content was calculated using the standard curve. A bovine serum albumin (BSA) standard solution was prepared by dissolving 100 mg of BSA in 100 mL of 0.05 M phosphate buffer (pH 7.8).

### 4.5. Soluble Sugar, Sucrose, Fructose, and Starch Content Measurement

Tissue samples (0.1 g each of root tips, new leaves, old leaves, and pseudostems) were extracted with 5 mL of 80% ethanol and incubated in a water bath for 30 min. The supernatant was collected and used to measure soluble sugar, sucrose, and fructose, while the residue was utilized for starch content determination.

#### 4.5.1. Soluble Sugar

An aliquot of 1 mL extract was diluted with 1 mL distilled water, and 0.5 mL anthrone ethyl acetate reagent (1 g anthrone in 50 mL ethyl acetate) was added. After adding 5 mL of concentrated ice-cold sulfuric acid, the solution was mixed and heated at 100 °C for 10 min. The sample was then cooled, and absorbance was measured at 630 nm against a blank control using a spectrophotometer (UV-1800, Shimadzu Corporation, Kyoto-Shi, Japan).

#### 4.5.2. Sucrose

For sucrose determination, 0.5 mL of the extract was combined with 0.5 mL of distilled water and 0.2 mL of 30% KOH, followed by incubation in a 95 °C water bath for 10 min. After cooling, 5 mL of anthrone–sulfuric acid reagent (2% anthrone in sulfuric acid) was added, and the mixture was incubated at 90 °C for 15 min. Absorbance was measured at 620 nm using a spectrophotometer (UV-1800, Shimadzu Corporation, Kyoto-Shi, Japan).

#### 4.5.3. Fructose

Fructose content was determined using the protocol of Yaphe and Arsenault [65]. The reaction mixture (0.4 mL extract, 0.6 mL 80% ethanol, 1 mL HCl, and 2 mL 0.1% resorcinol in 95% ethanol) was incubated at 80 °C for 10 min. After cooling, absorbance was recorded at 480 nm using a spectrophotometer (UV-1800, Shimadzu Corporation, Kyoto-Shi, Japan).

#### 4.5.4. Starch

Starch content was measured according to Han et al. [66]. After soluble sugar removal, the residue was treated with distilled water and HClO_4_ under ice bath conditions with intermittent shaking, followed by centrifugation. The starch content was quantified using a glucose standard curve using a spectrophotometer (UV-1800, Shimadzu Corporation, Kyoto-Shi, Japan).

### 4.6. Chlorophyll Fluorescence Measurement

Chlorophyll fluorescence was measured in mature leaves using a PAM-2500 portable fluorometer (Walz, Effeltrich, Germany). Leaves were dark-adapted for 15 min before being exposed to high-intensity light (650 nm, 3500 µmol photons m^−2^ s^−1^) for measurements of Fv/Fm and ETR. Each treatment was replicated five times.

### 4.7. Al and Nutrient Content Determination

After Al exposure for 24, 48, and 72 h, the seedlings were rinsed with distilled water and separated into root tips, new leaves, old leaves, and pseudostems. Samples were oven-dried (20 min at 105 °C, followed by drying at 70 °C), ground, and digested in HCl–HNO_3_ (3:1, *v*/*v*) using microwave-assisted digestion. Extracts were diluted to 50 mL and analyzed for Al, phosphorus (P), potassium (K), calcium (Ca), magnesium (Mg), iron (Fe), zinc (Zn), boron (B), and manganese (Mn) using inductively coupled plasma–optical emission spectroscopy (ICP-OES, Agilent 5110, Agilent Technologies, Santa Clara, CA, USA).

## 5. Statistical Analysis

Data were analyzed using two-way and three-way ANOVA to evaluate treatment effects using IBM SPSS (version 29.0). Prior to conducting ANOVA, the datasets were tested for normality (Shapiro–Wilk test) and homoscedasticity (Levene’s test). Significant differences among treatments (*p* < 0.05) were identified via Tukey’s honest significant difference (HSD) test. Data were presented as mean ± standard deviation (SD). Graphical visualizations, including bar charts and error bars, were produced using GraphPad Prism. Heatmaps and hierarchical clustering were created with Pheatmap (package version 1.0.12) in RStudio (version 2023.06.1), and correlation analyses were conducted using the corrplot package (package version 0.94).

## 6. Conclusions

The results revealed distinct patterns of Al accumulation between the two banana genotypes, Baodao (Al-sensitive) and Baxi (Al-tolerant), under varying levels of Al exposure. Root tips exhibited the highest Al accumulation, followed by pseudostems, while leaves showed the least accumulation. Al content increased with both exposure time and concentration, especially in the roots of Baodao, highlighting its greater sensitivity to Al stress due to excessive Al retention. In contrast, Baxi demonstrated more efficient Al translocation to aerial parts, suggesting a superior detoxification strategy via sequestration in the shoots. The enhanced activities of key enzymes, such as MDH and citrate synthase, in Baxi further indicate its more effective metabolic adjustment to Al stress, contributing to its greater tolerance.

Baxi displayed increased resilience under Al stress, maintaining higher levels of essential nutrients, particularly P, Zn, and Mg, thanks to its more efficient detoxification and nutrient transport mechanisms. On the other hand, Baodao experienced significant nutrient declines, particularly at higher Al concentrations (500 µM), which reflected its less effective response to Al toxicity. These findings suggest that Baxi possesses inherent mechanisms that confer greater tolerance to Al toxicity, making it a promising candidate for banana cultivation in Al-affected soils. This research highlights the potential for improving Al tolerance in banana cultivation, with Baxi’s Al-detoxifying strategies providing valuable insights for breeding and agricultural management.

## Figures and Tables

**Table 1:** Root elongation and relative root elongation of two banana genotypes under varying concentrations of aluminum (Al) at different exposure times.

Genotype	Al (µM)	Time (h)	Root Elongation (cm)	Relative Elongation of Root (%)
Baodao	0	24	0.38 ± 0.02 ^c^	100 ± 0.05 ^a^
		48	0.67 ± 0.01 ^b^	100 ± 0.08 ^a^
		72	0.95 ± 0.01 ^a^	100 ± 0.01 ^a^
	100	24	0.19 ± 0.01 ^e^	41.62 ± 0.021 ^b^
		48	0.28 ± 0.01 ^d^	40.94 ± 0.010 ^b^
		72	0.38± 0.01 ^c^	40.12 ± 0.008 ^b^
	500	24	0.07± 0.02 ^g^	17.82 ± 0.02 ^c^
		48	0.18± 0.02 ^f^	17.31 ± 0.02 ^c^
		72	0.17± 0.01 ^e^	17.68 ± 0.01 ^c^
Baxi	0	24	0.36 ± 0.01 ^c^	100 ± 0.01 ^b^
		48	0.62 ± 0.01 ^b^	100 ± 0.02 ^b^
		72	0.90 ± 0.01 ^a^	100 ± 0.02 ^b^
	100	24	0.38 ± 0.03 ^c^	107.50 ± 0.06 ^a^
		48	0.61 ± 0.01 ^b^	99.62 ± 0.01 ^b^
		72	0.87 ± 0.01 ^a^	97.52 ± 0.01 ^b^
	500	24	0.08 ± 0.04 ^f^	23.54 ± 0.05 ^c^
		48	0.13 ± 0.02 ^e^	21.81 ± 0.02 ^c^
		72	0.18 ± 0.01 ^d^	20.42 ± 0.01 ^c^
ANOVA (*F*-value)	Genotypes		534.33 ***	1025.38 ***
	Al		4212.16 ***	4827.71 ***
	Time		1744.14 ***	4.72 *
	Interaction		34.71 ***	1.41 ^ns^

Note: Each value represents an average of 3 replicates, with standard deviations shown. Different letters indicate significant differences at *p* ≤ 0.05 according to Duncan’s multiple range test, and * and *** denote significance at the 0.05 and 0.01 probability levels.

## Data Availability

The authors confirm that all experimental data are available and accessible via the main text and/or the supplemental data. The original contributions presented in this study are included in the article/Supplementary Material. Further inquiries can be directed to the corresponding author.

## Supplementary Materials

The following supporting information can be downloaded at: https://www.mdpi.com/article/10.3390/plants14030385/s1, Table S1. The P and Zn Content in Tissues of Baodao and Baxi after 24, 48, and 72 h of Exposure to 0, 100, and 500 µM Al. Table S2. The Mg and Mn Content in Tissues of Baodao and Baxi after 24, 48, and 72 h of Exposure to 0, 100, and 500 µM Al. Table S3. The Fe and K Content in Tissues of Baodao and Baxi after 24, 48, and 72 h of Exposure to 0, 100, and 500 µM Al. Table S4. The B and Ca Content in Tissues of Baodao and Baxi after 24, 48, and 72 h of Exposure to 0, 100, and 500 µM Al.
