# Supplementary material for: Metabolic and Nutritional Responses of Contrasting Aluminium-Tolerant Banana Genotypes Under Al Stress
Source: Plants (Basel). 2025 Jan 27;14(3):385. doi: 10.3390/plants14030385 (PMC11820201; doi:10.3390/plants14030385)
Supplement: Supplementary file 1 [file plants-14-00385-s001.zip › plants-3402166-supplementary.pdf]

Table S1. The P and Zn Content in Tissues of Baodao and Baxi After 24, 48, and 72 Hours of Exposure to 0, 100, and 500 μM Al.

Genotype	Al (μM)	Time (h)	P (mg/g DW) Root	P New leaves	P Old leaves	P Pseudostem	Zn (μg/g DW) Root	Zn New leaves	Zn Old leaves	Zn Pseudostem
Baodao	0	24	2.72^{a}	3.75^{ab}	1.74^{bc}	2.38^{a}	805.2^{d}	36.9^{ab}	47.81^{a}	568.87^{a}
	100	24	2.36^{ab}	5.34^{a}	2.68^{a}	3.03^{a}	1740.6^{bc}	92.93^{a}	38.95^{ab}	323.94^{abc}
		48	2.00^{ab}	3.46^{ab}	1.98^{b}	2.74^{a}	2121.3^{ab}	21.00^{ab}	53.25^{a}	249.16^{bc}
		72	2.70^{a}	2.98^{b}	1.48^{b-d}	2.94^{a}	2877.2^{a}	59.85^{ab}	38.12^{ab}	410.76^{ab}
	500	24	1.34^{b}	2.51^{b}	1.27^{cd}	1.23^{b}	1075.6^{cd}	32.41^{ab}	14.73^{bc}	137.30^{c}
		48	2.03^{ab}	2.85^{b}	1.07^{cd}	1.40^{b}	1093.5^{cd}	16.25^{b}	7.94^{c}	305.83^{abc}
		72	1.77^{ab}	3.22^{ab}	0.88^{d}	0.99^{b}	1058.4^{cd}	54.67^{ab}	6.70^{c}	333.37^{abc}
Baxi	0	24	3.68^{bc}	2.76^{b}	1.53^{bc}	2.26^{bc}	880.7^{c}	25.88^{b}	33.57^{ab}	467.29^{a}
	100	24	5.55^{ab}	8.52^{a}	8.19^{a}	4.29^{a}	1866.8^{bc}	99.32^{a}	79.34^{a}	399.74^{a}
		48	5.45^{ab}	4.20^{b}	3.91^{b}	3.51^{ab}	4001.4^{a}	52.65^{ab}	60.53^{ab}	252.77^{a}
		72	5.78^{a}	2.64^{b}	2.74^{bc}	2.89^{abc}	3877.1^{a}	48.62^{b}	69.81^{a}	577.29^{a}
	500	24	1.98^{c}	4.29^{b}	2.22^{bc}	2.66^{abc}	1353.6^{c}	32.29^{b}	67.87^{a}	414.31^{a}
		48	3.29^{c}	3.50^{b}	1.21^{c}	2.14^{bc}	3173.6^{ab}	57.93^{ab}	50.32^{ab}	396.04^{a}
		72	3.76^{bc}	3.80^{b}	1.09^{c}	1.36^{c}	1794.5^{bc}	50.92^{ab}	10.78^{b}	240.00^{a}
ANOVA (*F*-value)	Genotype (G)		48.77***	0.51^{ns}	13.66***	4.40*	11.07**	0.12^{ns}	4.51*	0.23^{ns}
	Al		12.65***	4.61*	5.50*	18.11***	27.49***	3.25^{ns}	6.02**	6.41**
	Time (T)		1.04^{ns}	4.96*	8.49**	1.79^{ns}	4.47*	1.08^{ns}	1.06^{ns}	0.53^{ns}
	G◊Al		7.33**	2.63^{ns}	11.37***	1.84^{ns}	2.21^{ns}	0.52^{ns}	4.32*	1.58^{ns}
	G◊T		0.22^{ns}	1.90^{ns}	3.41*	1.08^{ns}	2.68^{ns}	0.76^{ns}	0.66^{ns}	0.36^{ns}
	Al◊T		0.89^{ns}	3.83*	4.60**	0.55^{ns}	2.37^{ns}	1.69^{ns}	0.68^{ns}	1.20^{ns}
	G◊Al◊T		0.29^{ns}	0.57^{ns}	2.04^{ns}	0.30^{ns}	0.71^{ns}	0.21^{ns}	0.72^{ns}	0.73^{ns}

Each value represents the mean ± standard deviation of three replicates. Different letters indicate significant differences at $p \leq 0.05$, determined by Duncan's multiple range test. *, **, and *** denote significant differences at $p \leq 0.05$, $p \leq 0.01$, and $p \leq 0.001$, respectively.

Table S2. The Mg and Mn Content in Tissues of Baodao and Baxi After 24, 48, and 72 Hours of Exposure to 0, 100, and 500 μM Al.

Genotype	Al (μM)	Time (h)	Mg (mg/g DW) Root	Mg New leaves	Mg Old leaves	Mg Pseudostem	Mn (μg/g DW) Root	Mn New leaves	Mn Old leaves	Mn Pseudostem
Baodao	0	24	2.62^{a}	1.76^{bc}	1.91^{a}	1.19^{c}	28.09^{bc}	42.52^{a}	34.57^{bc}	29.59^{a}
	100	24	2.25^{ab}	2.42^{a}	1.17^{bc}	3.44^{a}	54.90^{ab}	36.07^{ab}	19.95^{c}	16.87^{bc}
		48	2.21^{ab}	0.98^{c}	1.62^{ab}	1.64^{bc}	33.46^{bc}	22.77^{bc}	30.71^{bc}	17.39^{abc}
		72	2.53^{a}	1.46^{bc}	1.06^{cd}	2.46^{ab}	67.53^{a}	19.25^{bc}	32.13^{bc}	26.73^{ab}
	500	24	0.96^{c}	2.01^{ab}	0.58^{de}	1.71^{bc}	32.50^{bc}	8.57^{c}	56.70^{a}	10.57^{c}
		48	1.65^{abc}	1.43^{bc}	0.48^{e}	1.18^{c}	34.14^{bc}	8.49^{c}	18.51^{c}	15.64^{bc}
		72	1.29^{bc}	1.86^{abc}	1.47^{abc}	0.81^{c}	25.88^{c}	10.38^{c}	48.28^{ab}	9.80^{c}
Baxi	0	24	1.95^{bc}	1.07^{b}	2.53^{ab}	1.68^{a}	37.43^{b}	39.31^{a}	83.20c	35.63^{a}
	100	24	2.73^{b}	4.82^{a}	3.08^{a}	2.10^{a}	75.80^{a}	46.91^{a}	118.72^{bc}	40.64^{a}
		48	4.48^{a}	1.73^{b}	2.02^{abc}	1.89^{a}	88.56^{a}	34.71^{a}	88.64^{bc}	38.29^{a}
		72	3.87^{a}	1.18^{b}	1.44^{bc}	1.68^{a}	77.58^{a}	23.46^{a}	120.31^{bc}	25.48^{a}
	500	24	1.49^{c}	2.03^{b}	1.58^{abc}	2.65^{a}	37.05^{b}	37.68^{a}	179.16^{b}	30.91^{a}
		48	2.04^{b}	2.02^{b}	0.62^{c}	1.46^{a}	44.98^{b}	23.92^{a}	117.63^{bc}	18.04^{a}
		72	1.73^{bc}	1.84^{b}	0.82^{c}	1.36^{a}	27.85^{b}	29.29^{a}	394.30^{a}	29.08^{a}
ANOVA (*F*-value)	Genotype (G)		5.84*	0.43^{ns}	8.93**	0.52^{ns}	10.25**	5.48*	98.15***	10.81**
	Al		24.10***	5.16*	16.31***	5.12*	23.38***	10.18***	20.27***	5.15*
	Time (T)		2.83^{ns}	9.99***	0.96^{ns}	3.63*	0.00^{ns}	1.60^{ns}	9.51***	0.08^{ns}
	G◊Al		10.11***	5.69**	1.32^{ns}	3.30*	2.43^{ns}	3.36*	15.59***	0.61^{ns}
	G◊T		0.53^{ns}	1.59^{ns}	2.89^{ns}	0.20^{ns}	1.40^{ns}	0.19^{ns}	6.75**	0.58^{ns}
	Al◊T		0.82^{ns}	7.62***	1.45^{ns}	1.10^{ns}	0.80^{ns}	0.95^{ns}	7.12***	0.06^{ns}
	G◊Al◊T		1.27^{ns}	1.64^{ns}	0.87^{ns}	0.81^{ns}	0.83^{ns}	0.16^{ns}	5.78**	1.02^{ns}

Each value represents the mean ± standard deviation of three replicates. Different letters indicate significant differences at $p \leq 0.05$, determined by

Duncan's multiple range test. *, **, and *** denote significant differences at $p \leq 0.05$, $p \leq 0.01$, and $p \leq 0.001$, respectively.

Table S3. The Fe and K Content in Tissues of Baodao and Baxi After 24, 48, and 72 Hours of Exposure to 0, 100, and 500 μM Al.

Genotype	Al (μM)	Time (h)	Fe (μg/g DW)				K (mg/g DW)			
			Root	New leaves	Old leaves	Pseudostem	Root	New leaves	Old leaves	Pseudostem
Baodao	0	24	1024.21^{abc}	147.25^{a}	143.40^{a}	104.38^{b}	45.19^{ab}	64.39^{a}	52.22^{a}	77.07^{ab}
	100	24	1629.85^{a}	108.91^{ab}	53.24^{bc}	145.82^{a}	31.40^{ab}	65.95^{a}	29.40^{bc}	47.95^{bc}
		48	1177.32^{abc}	80.67^{ab}	62.80^{bc}	70.87^{bc}	36.49^{ab}	45.52^{a}	43.00^{ab}	49.80^{bc}
		72	1330.94^{ab}	70.39^{ab}	37.25^{c}	78.30^{bc}	57.21^{a}	65.87^{a}	43.35^{ab}	82.15^{a}
	500	24	749.91^{bc}	57.85^{ab}	41.05^{c}	15.28^{d}	22.55^{b}	57.56^{a}	21.99^{cd}	30.95^{c}
		48	1441.62^{a}	123.11^{ab}	78.22^{b}	20.70^{d}	31.62^{ab}	63.71^{a}	11.83^{d}	49.82^{bc}
		72	636.31^{c}	47.45^{b}	31.42^{c}	46.76^{cd}	27.77^{b}	68.36^{a}	22.53^{cd}	53.79^{abc}
Baxi	0	24	1062.70^{c}	133.35^{ab}	104.57^{ab}	166.08^{a}	43.75^{b}	38.22^{b}	58.50^{b}	65.71^{ab}
	100	24	1989.02^{bc}	162.30^{a}	141.41^{a}	99.88^{ab}	66.27^{ab}	139.23^{a}	109.43^{a}	40.15^{b}
		48	4993.78^{a}	151.30^{a}	64.49^{bcd}	79.02^{ab}	91.38^{a}	74.01^{b}	59.22^{b}	45.65^{b}
		72	2706.56^{b}	42.5^{c}	39.01^{cd}	67.16^{ab}	96.02^{a}	70.31^{b}	34.21^{b}	109.23^{a}
	500	24	1772.44^{bc}	77.2^{bc}	84.07^{abc}	35.03^{b}	26.12^{b}	70.33^{b}	51.29^{b}	64.42^{b}
		48	1873.81^{bc}	148.0^{a}	78.83^{abcd}	51.29^{b}	40.78^{b}	64.91^{b}	22.00^{b}	62.11^{b}
		72	968.78^{c}	83.2^{bc}	14.73^{d}	60.62^{ab}	56.39^{ab}	79.72^{b}	14.04^{b}	48.50^{b}
ANOVA (*F*-value)	Genotype (G)		22.38***	1.32^{ns}	0.00^{ns}	99.03^{ns}	9.28**	0.74^{ns}	7.23*	0.15^{ns}
	Al		20.02***	5.46**	26.39***	17.74***	7.86**	4.70*	12.79***	3.54*
	Time (T)		5.28**	3.76*	5.90**	0.29^{ns}	2.13^{ns}	1.42^{ns}	3.06^{ns}	3.36*
	G◊Al		9.37***	1.24^{ns}	6.04**	2.87^{ns}	4.63*	6.73**	1.54^{ns}	1.44^{ns}
	G◊T		2.98^{ns}	0.45^{ns}	3.40*	0.22^{ns}	0.26^{ns}	1.09^{ns}	4.81*	0.08^{ns}
	Al◊T		2.08^{ns}	2.44^{ns}	2.47^{ns}	1.12^{ns}	0.61^{ns}	1.87^{ns}	0.96^{ns}	3.40*
	G◊Al◊T		4.49**	0.70^{ns}	1.05^{ns}	0.15^{ns}	0.31^{ns}	0.94^{ns}	1.88^{ns}	1.07^{ns}

Each value represents the mean ± standard deviation of three replicates. Different letters indicate significant differences at $p \leq 0.05$, determined by Duncan's multiple range test. *, **, and *** denote significant differences at $p \leq 0.05$, $p \leq 0.01$, and $p \leq 0.001$, respectively.

Table S4. The B and Ca Content in Tissues of Baodao and Baxi After 24, 48, and 72 Hours of Exposure to 0, 100, and 500 μM Al.

Genotype	Al (μM)	Time (h)	B (μg/g DW)				Ca (mg/g DW)			
			Root	New leaves	Old leaves	Pseudostem	Root	New leaves	Old leaves	Pseudostem
Baodao	0	24	41.60^{a}	38.39^{a}	30.82^{b}	26.81^{ab}	3.50^{a}	6.66^{a}	9.65^{a}	4.37^{b}
	100	24	44.36^{a}	25.65^{ab}	80.12^{a}	34.10^{a}	2.65^{ab}	6.52^{a}	4.11^{bc}	7.79^{a}
		48	28.27^{a}	25.52^{ab}	38.51^{ab}	18.27^{ab}	1.35^{bc}	5.27^{ab}	5.39^{b}	4.26^{b}
		72	42.11^{a}	26.04^{ab}	42.39^{ab}	23.33^{ab}	1.50^{bc}	3.29^{b}	4.71^{bc}	7.64^{a}
	500	24	35.59^{a}	14.93^{ab}	40.42^{ab}	17.92^{ab}	2.00^{abc}	4.61^{ab}	2.38^{cd}	1.52^{c}
		48	31.29^{a}	9.92^{b}	38.25^{ab}	6.97^{b}	0.85^{bc}	4.38^{ab}	1.53^{d}	2.26^{bc}
		72	42.11^{a}	12.05^{b}	12.01^{b}	10.06^{b}	0.31^{c}	3.19^{b}	1.58^{d}	2.66^{bc}
Baxi	0	24	32.28b	26.76^{b}	26.76	28.73^{a}	4.01^{a}	4.77^{b}	11.90^{b}	4.26^{a}
	100	24	62.51^{ab}	115.32^{a}	38.84^{c}	22.95^{a}	3.09^{ab}	14.40^{a}	28.71^{a}	5.88^{a}
		48	91.21^{a}	79.98^{ab}	209.53^{a}	35.11^{a}	2.48^{ab}	6.77^{b}	5.95^{bc}	3.33^{a}
		72	94.88^{a}	96.61^{a}	82.26^{bc}	30.69^{a}	4.01^{ab}	6.48^{b}	7.08^{bc}	5.00^{a}
	500	24	35.87^{b}	28.94^{b}	129.40^{b}	26.88^{a}	2.52^{ab}	6.37^{b}	11.14^{b}	3.11^{a}
		48	40.84^{b}	27.47^{b}	44.76^{c}	15.02^{a}	2.03^{ab}	5.55^{b}	1.64^{c}	3.19^{a}
		72	50.80^{b}	24.83^{b}	36.74^{c}	26.63^{a}	1.43^{b}	2.95^{b}	2.71^{c}	3.65^{a}
ANOVA (*F*-value)	Genotype (G)		10.52**	13.05***	16.06***	2.09^{ns}	6.00*	3.65*	38.73***	0.41^{ns}
	Al		11.52***	12.87***	10.71***	2.93^{ns}	16.65***	7.27**	31.64***	17.75***
	Time (T)		0.91^{ns}	0.35^{ns}	8.85***	0.40^{ns}	1.93^{ns}	7.71**	19.08***	2.41^{ns}
	G◊Al		12.25***	12.67***	3.45*	0.48^{ns}	0.19^{ns}	9.83***	7.39**	4.71*
	G◊T		1.66^{ns}	0.20^{ns}	3.10^{ns}	0.54^{ns}	0.22^{ns}	1.85^{ns}	19.42***	0.18^{ns}
	Al◊T		0.33^{ns}	0.21^{ns}	2.68*	0.27^{ns}	0.65^{ns}	3.46*	6.72***	2.71*

G◊Al◊T	0.84^{ns}	0.27^{ns}	0.81^{ns}	0.50^{ns}	0.06^{ns}	1.21^{ns}	8.19^{***}	0.21^{ns}

Each value represents the mean ± standard deviation of three replicates. Different letters indicate significant differences at $p \leq 0.05$, determined by Duncan's multiple range test. *, **, and *** denote significant differences at $p \leq 0.05$, $p \leq 0.01$, and $p \leq 0.001$, respectively.
